# “I am not telling. The mobile is telling”: Factors influencing the outcomes of a community health worker mHealth intervention in India

**DOI:** 10.1371/journal.pone.0194927

**Published:** 2018-03-27

**Authors:** Onaedo Ilozumba, Marjolein Dieleman, Nadine Kraamwinkel, Sara Van Belle, Murari Chaudoury, Jacqueline E. W. Broerse

**Affiliations:** 1 Athena Institute, Vrije Universiteit, Amsterdam, The Netherlands; 2 Department of Public Health, Institute of Tropical Medicine, Antwerp, Belgium; 3 NEEDS NGO, Deoghar, India; University College London, UNITED KINGDOM

## Abstract

**Introduction:**

Improving maternal health outcomes remains a priority in Low and Middle Income Countries. With the rapid proliferation of mobile health technologies, there is an increased interest in understanding how these technologies can effectively improve maternal health outcomes particularly maternal health seeking knowledge and behaviors. However, few studies present clear explanations of the program developers’ rationale (theory of change) and contextual factors that could influence program outcomes. This mixed-methods study assesses Mobile for Mothers, a community health workers (CHW) utilized maternal mHealth intervention. We present the program developers’ rationale and utilize it as a framework to guide our study that aimed to identify intervention-related and contextual factors, which influence the observed outcomes of a CHW, utilized mHealth intervention.

**Materials and methods:**

Quantitative methods (a questionnaire with 740 women who received the intervention and survey of 57 CHWs who utilized the intervention) and qualitative methods (12 interviews and 4 group discussions with CHWs and 20 interviews and 5 group discussions with pregnant and lactating women and 15 interviews and 2 group discussions with men) were conducted. These were used to understand how the mHealth intervention was implemented and to gain insight into contextual factors that potentially influenced the observed intervention outcomes.

**Results:**

Results were grouped following three categories: (1) perceptions and experiences of CHWs utilizing the mHealth technology; (2) CHW-related outcomes; and (3) contextual factors that influence maternal health-seeking behavior. The overall response of CHWs and community members to the intervention was positive. However, contextual factors like the relationship between the CHWs and their respective communities, the pregnant women’s decision-making power and lack of access due to financial influenced the observed outcomes.

**Conclusion:**

Mobile health applications are promising interventions for improving the performance of CHWs and health-seeking behavior of pregnant women. However, the contextual factors play a crucial role in intervention outcomes and need to be explicated by program developers during intervention design and implementation.

## Introduction

In Low and Middle-income countries (LMICs), like India, Community Health Worker (CHW) programs have been introduced to address the common challenge of health worker shortages (or human resources for health). CHWs work within multiple health domains which frequently include maternal, neonatal and child health [1].There is substantial evidence that integrating CHWs into maternal and child health service delivery has positive effects on key indicators, such as the uptake of antenatal care, the delivery by a skilled health professional and receipt of vaccinations for under-five children [1,2]. However CHWs performance outcomes are hampered by factors such as limited training, which result in low knowledge and skills levels and poorly equipped facilities [3].

Interventions aimed at improving maternal health knowledge, skills, and ultimately, services delivered by CHWs have often introduced job aids to improve their performance and output. The increased access to mobile phones—95% of the global population currently being covered by a mobile network—has led to the utilization of mobile health (mHealth) tools as an intervention to improve CHWs’ knowledge, skills and performance [4]. Many definitions of mHealth exist. We use here Istepanian et al (2004), who define mHealth as “mobile computing, medical sensor, and communications technologies for health care” [5], and there are a broad range of mHealth interventions with various functionalities from data collection to knowledge and training functions [6].

A few studies have indicated that with adequate training CHWs can learn to operate mobile phones in the context of health care provision, have positive perceptions of the intervention and consider them useful in improving service delivery and performance [7]. The utilization of mHealth applications has been linked to outcomes such as improved motivation, empowerment, self-efficacy and health knowledge amongst CHWs [8,9]. Chib et al (2008) found that such interventions could be perceived to improve CHW credibility within their community. However, it is important to note that a significant portion of this evidence is not specific to the use of applications on mobile phones or tablets and is related more generally to mobile phone use, including text messaging and voice calls. Much less is known about CHWs utilization and perception of mHealth applications, i.e. the software designed to run on a mobile phone [10]. The aim of this paper is to identify intervention-related and contextual factors that influence the observed outcomes of a CHW utilized mHealth intervention in India.

India presents an interesting case study for the use of mHealth among CHWs for maternal health for several reasons, including the maternal mortality burden and a longstanding CHW system. Maternal mortality in India remains one of the worst in the world, accounting for approximately 15% of global maternal deaths according to the latest estimates by the World Health Organization (WHO) [11]. In 2015 the National Rural Health Mission (NRHM) was created to address the marked differences in health indicators between rural and urban communities, particularly for women and children (7). One of the adopted strategies was the Accredited Social Health Activists (ASHAs) program, which attempted to address the shortage in health staff. In the time since program initiation (2005) there is evidence to suggest that ASHAs have been influential in the improvement of health indicators particularly related to maternal and child health, including attendance of antenatal care (ANC), institutional delivery, and childhood vaccinations (8).

Literature on maternal health-seeking behavior has identified several factors related to poor maternal health outcomes; some of these are applicable to LMICs in general while others are specific to the Indian context. Many of these factors can be addressed by referencing the Three Delays model [12]. The first delay, failing to decide on receiving appropriate care is well researched within the Indian context, with studies showing that low knowledge and education levels are associated with sub-optimal maternal health-seeking behavior [13]. In situations where women may have good maternal health knowledge and a positive attitude towards health seeking, they are often, as in many LMIC settings, not the sole decision-maker regarding their health-seeking behavior, and are strongly influenced by husbands or (mother) in-laws [14,15]. The second delay, women’s ability to reach the health facility, has received attention from national and state-level governmental institutions, and local non-governmental organizations (NGOs) in India. The Mamta Vahan scheme, implemented at national level, is an ambulance service for pregnant women and was introduced to reduce transportation barriers that prevent women from reaching health facilities. Some work has also been done to address financial barriers associated with maternal health seeking; these influence both women’s decision to seek care and their ability to access the health institution. The Janani Suraksha Yojana (JSY) and Janani Shishu Suraksha Karyakram (JSSK) schemes were implemented on a national scale in 2005 and 2011 respectively. Through these schemes, pregnant women receive free healthcare services, including ANC, delivery and postnatal care (PNC) [16]. However, evidence suggests that implementation barriers, such as the mode of payment, e.g. direct bank transfer, affect the effectiveness of these schemes [16,17]. When women are able to overcome the first two delays and reach the health facility, the third delay could come into play, i.e. the quality of care women receive at the health facility. Quality of healthcare in India, as in many other LMIC settings, is negatively affected by many factors, including health worker shortages and poor availability of equipment and medicines. Previous research indicates that quality of care particularly in rural or slum communities is often poor, with evidence of disrespect for patients, corruption and poorly equipped facilities [18]. Additional information on the ASHA, Mamta Vahan, Janani Suraksha Yojana (JSY) and Janani Shishu Suraksha Karyakram schemes can be found in Table 1.

**Table 1:** Maternal health provision schemes (source nation health mission and center for innovations in public systems).

Program	Program Description
Accredited Social Health Activist (ASHA)	The Indian Ministry of Health had also introduced female CHWs in 2005 in rural communities under the National Rural Health Mission (NRHM). On national level, they are called Accredited Social Health Activists (ASHAs), and in Jharkhand India where MfM was implemented, Sahiyyas. Some selection criteria for ASHA’s include - They must be a female resident of the village preferably in the age group of 25 to 45 years. - Preferably have up to standard 10 education - Capacity building of ASHA should be a continuous process which involves multiple training sessions to acquire the necessary knowledge, skills and confidence. - The ASHAs receive performance-based incentives for promoting universal immunization, referral and escort services for Reproductive & Child Health (RCH) and other healthcare programs, and construction of household toilets.
Janani Shishu Suraksha Karyakaram (JSSK)	JSSK was launched on the 1st June, 2011. The scheme has two levels of entitlements for pregnant women and also for newborns and infants. **For pregnant women:** Free and cashless delivery and exemption from user charges. Free C-Section. Free drugs, consumable and diagnostics. Free diet during stay in the health institutions. Free provision of blood. Free transport from home to health institutions. Free transport between facilities in case of referral. Free drop back from Institutions to home after 48hrs stay **For sick newborns and infants:** Free treatment. Free drugs and consumables. Free diagnostics. Free provision of blood. Exemption from user charges. Free transportation to and from health institutions
Janani Suraksha Yojana (JSY)	Janani Suraksha Yojana (JSY) was launched in April 2005 and promotes institutional delivery among poor pregnant women and has a special focus on Low Performing States (LPS). LPS are states with 25% or less were termed, Jharkhand the intervention state is included. It involves the provision of cost assistance to both the woman who delivers at the health care facility and her ASHA. In rural LPS, the woman would recieve1400 rupees and her ASHA would receive 600 rupees per institutional delivery
Mamata Vahan Scheme	Mamata Vahan is a part of the NRHM ambulances/ patient transport vehicles which primarily transports pregnant women to government hospitals for delivery and back home afterwards. They can also take women to referral hospitals, primary health centers or community health centers depending upon the medical requirement. In Jharkhand a person who owns a four-wheeler (defined as a vehicle which a pregnant woman can lie down inside) can register the vehicle as a 'Mamata Vahan' with the respective Civil Surgeon's office. The office of Civil Surgeon runs a call center and pregnant women or their Sahiyya can call this call center to be connected to a Mamata Vahan driver. They can also call the cellphone of the Mamata Vahan driver to request attention. The services of the Mamata Vahan are provided free of charge to the women.

In addition to these known factors that influence maternal health seeking, it is also crucial to understand that the mHealth interventions are frequently implemented within the context of an existing intervention and a health system. Overall implementation of the ASHA program could thus potentially influence the outcomes of the mHealth intervention. Limited trust of the AHSAs by the community, the low educational attainment of the ASHAs and disfranchisement or discontent with working conditions of the ASHAs, could potentially affect the outcomes of the mHealth intervention [19,20]. In a similar way, existing infrastructural and local health system level problems (human resources, infrastructure, drug supply issues) could also be limiting or enabling factors to mHealth intervention effectiveness. Overall, these factors point to the complexity of introducing mHealth technology within the Indian maternal healthcare and ASHA system.

While there is growing evidence for the impact of maternal mHealth in LMICs, Lee et al.’s (2016) review highlighted shortcomings. There is still limited data on mHealth interventions in maternal, newborn and child health in South Asia with the majority of programs occurring in sub-Saharan African and East Asia. Of the reported programs few explained their program rationale and were under-theorized with no discussion of mechanisms and impacts [21]. This paper proposes to address some of these gaps by assessing Mobile for mothers, a CHW-utilized maternal mHealth intervention. We present the program developers rationale (theory of change) and utilize it as a framework to guide our examination of intervention-related and contextual factors, which could influence observed outcomes of a CHW-utilized maternal mHealth intervention.

## Intervention background

### Intervention developers rationale

Mobile for Mothers (MfM), a software application, was conceptualized and initiated in 2011 by NEEDS, an Indian non-governmental organization (NGO), and SIMAVI, a Dutch NGO. A sub-district level study revealed that approximately 27% of deaths recorded among women in the district were preventable maternal deaths. Low maternal health knowledge levels among CHWs (AHSAs within the NHRM system and locally called Sahiyyas) and the target group (pregnant and lactating women), and poor health-seeking behavior of pregnant and lactating women were identified by NEEDS and SIMAVI as possible causes. MfM was developed as a potential solution to these problems.

At the start of the MfM intervention, representatives from NEEDS, SIMAVI and government health workers from Jharkhand outlined three main intervention objectives: (1) to improve the information and referral services of CHWs to pregnant women in rural India; (2) to increase the knowledge level and health-seeking behavior among women living in the intervention sub-district; and (3) to improve the collection of key health indicators related to safe motherhood at sub-district level, which would potentially help district level authorities to better orient district health policy.

### Intervention design

MfM is a mobile application that is installed on a standard mobile phone. The application included a multimodal components text, image and audio, which presented information in Hindi. The mobile phone was pre-loaded with phone credit and provided free of charge to 376 CHWs. The recruited CHWs received a two-day training about the modules on the mobile phone and how to apply the application for health education and data collection. CHWs then utilized the MfM application during routine home educational visits with pregnant and lactating women in their communities.

The MFM application consisted of four modules: (1) registration of the pregnant woman, (2) antenatal care (ANC), (3) intra-natal care (INC) i.e. delivery at a healthcare facility or with a skilled health professional, and (4) postnatal care (PNC). The MfM database was stored on a cloud server and contained all collected information from the MfM phones.

### Evaluation results of MfM

MfM was designed as a quasi-experimental study with three groups: (1) an intervention group that received MfM; (2) a quasi-control group that received NGO programs; and (3) a control group that only received standard government programs. All groups received standard government programs that included the recruitment and support of CHWs who conducted home visits to the pregnant and lactating women, the Mahatma Van scheme, which made ambulances available, and the National Population Stabilization Fund (JSK) scheme, which provided financial incentives for women delivering at the hospital. The quasi-control group, in addition to the standard government offerings, also received targeted interventions from NEEDS, including the provision of maternal health education messages amongst others through dramas and dance, and programs geared at increasing male involvement in maternal health.

In a quantitative evaluation, presented in another article we evaluated three key outcomes: (1) maternal health knowledge, (2) attendance of four or more ANC, and (3) delivery at a health facility/with a skilled health professional. For each outcome we compared results between three groups of women, those who had received the MfM intervention, those who received other NEEDS interventions excluding MfM (quasi-control) and those who received only the standard care government interventions (control group). We found that women who were in the MfM group who received the mobile intervention had higher maternal health knowledge (71.2% vs 58.5% and 50.6%) and were more likely to attend four or more ANC visits (47.8% vs 27.1 and 17.3%) and deliver their babies at a health facility (77.6% vs 60.3% vs 48.2%) than those in the quasi control and control groups (p<0.05). However, using ANC as an example, we note that even though the women in the intervention group were the highest performing, their indicators remained suboptimal when compared to initial program designers targets (Table 1) and global recommendations of four or more antenatal care visits [11]. Only half of the women in the study area attended 4 or more ANC visits and about three out of four women delivered at a health facility. These results suggest that there are other factors at work affecting the observed MfM outcomes

### Theory of change

In the development of the intervention the program designers, NEEDS and SIMAVI, put forth what they referred to as a Theory of Change (see Table 2). A Theory of Change can be defined as the implicit assumptions of programme designers [22]. They assumed that training, follow-up and supervision of CHWs who received the MfM phones, would lead to increased knowledge among the CHWs and pregnant women. This knowledge increase would in its turn result in improved maternal health-seeking behaviors and ultimately, in reduced maternal and neonatal mortality. Although the inputs and activities during implementation adhered to the program designers’ theory of change, the observed outcomes differed from program designers’ expectations. The Theory of Change of the programme designers guided the development of the study research questions and study analysis. We sought to understand what intervention-related and contextual factors influenced the observed intervention outcomes. This is important as it allows for an exploration of factors that influenced the observed outcomes of how the mHealth intervention worked in this context.

**Table 2:** Theory of change (source: NEEDS and SIMAVI).

Hierarchy	Program Causal Chain of mobile for mother’s health
**Impact**	• Accelerated reduction in MMR and Neonatal death rate. • Policy impact of MfM: Modification in the design/implementation of NRHM at the state level through lessons learned from NEEDS experience.
**Outcome**	• Intended behavioral changes in target groups (mothers) with regard to seeking institutional services for health care, delivery, pre-and-post natal care. ○ 80% of pregnant women attend 4 ANC, have a birth preparedness plan anddeliver at a health institution or with the assistance of a skilled birth attendant ○ 60% receive two PNC visits
**Output**	• Improved knowledge of the target group and CHWs on health behavior during pregnancy till 28 days after the delivery
**Activities**	• Training of CHWs, and follow-up, supervision. • System strengthening in terms implementation, capacity-building of health managers and supply chain management • Case Management
**Inputs**	• Funds, materials, human resources, technical assistance.

### Research objectives

In light of the evaluation results and using the program developers’ Theory of Change as a guide, we aimed to identify intervention-related and contextual factors potentially influencing observed outcomes of the intervention through primary qualitative and quantitative data collection from CHWs, women and men who were involved with the MfM intervention. Although the mHealth application was utilized by the CHWs, other stakeholders such as the pregnant women and men in the community are important in understanding the intervention outcome. We hypothesized that there were multiple pathways that influenced MfM outputs and outcomes:

- As the MfM was administered by CHWs, the characteristics of these health workers and their relationship with the pregnant women could have influenced their acceptance and utilization of the application.
- Furthermore the application was used within a specific context, with its own dynamics that would also have influenced program output and outcomes. Examples are the existing relationships between CHWs and community members, and health-seeking behavior and provider behavior influenced by community-health system relations, community-provider relationships, socio-cultural factors, literacy, level of poverty, vulnerable groups within the population, power relations within community, within household, institutional trust, social capital, community trust, community leadership.
- Level of the local health system: provider attitudes, management and supervision, trust in the health system, health system-community relations, pluralistic health system (presence of private sector, traditional healers), transport and access, financial barriers.

In order to reach our research aim, we formulated the following research questions:

1. What factors serve as facilitators or barriers to CHW acceptance and utilization of MfM?
2. What were CHWs perceptions of MfM on their personal knowledge and performance?
3. What existing factors, contextual or otherwise, could explain the observed patterns in maternal health seeking behavior among women who received the MfM intervention?

## Methodology

A mixed methods approach was adopted to explore the contextual factors related to the observed outcomes of a CHW-utilized maternal mHealth intervention in rural India. This mixed methods assessment included household surveys of community women who received the intervention, a survey among CHWs who utilized the intervention and qualitative interviews with CHWs, pregnant and lactating women and men in the community. Quantitative and qualitative data collection occurred concurrently with the intention of data triangulation to enhance validity

### Ethics statement

This study was part of a larger study, Mobile for Mothers, and was approved by the Institutional Review Board, Center for Media Studies in New Delhi. Informed consent to participate was gathered verbally from the participants according to the principles of meaningful consent; corresponding to the participants native tongue and the communication of the purpose, methods, risks and benefits of this study on the basis of their world view and value system [23]. Verbal consent was considered most appropriate due to the low literacy levels among the study population and was approved by the ethical board. For the purposes of record keeping, when participants indicated verbally that they were willing to participate in the interview, the interviewer would themselves date and sign the informed consent form that had been read out to the participants.

### Study setting

All data collection occurred in Sarwan block, Deoghar, Jharkhand State in India from April 2015 to November 2016. Jharkhand is a predominantly rural state in eastern India and is located between the states of Bihar and West Bengal. Deoghar is situated 253 kilometers from Patna, Bihar, and has a population of 203,116 people with 82.68% living in rural area [24]. According to the census, the average literacy is 64.85% with female literacy being 51.80%, and the predominant religion in Deoghar is Hinduism (78.09%). Furthermore, Jharkhand experiences relatively high maternal mortality ratio of 219 per 100.000 live births, which is higher than the national average of 179 per 100.000 live births [25].

### Data collection

An overview of all participants included in the study is found in Table 3

**Table 3:** Study sample.

Methodology	Group	Number of participants (total n)
Quantitative Surveys		
Household Survey	Pregnant and lactating women	740
Community Health Worker Survey	Community health workers	57
Qualitative Interviews		
Semi-structured individual interviews	Men Women CHWs	15,20,12
Group interviews	Women (n = 5 groups) Men (n = 2 groups) CHWs (n = 4 groups)	12 (2–3 per group) 16 (6 and 10 in each) 16 (4 in each)

#### Quantitative surveys

Quantitative data was obtained from women who had participated in the study through household sampling. Respondents were selected from primary sampling units (villages), in which PPS (Probability Proportional to Size) sampling principles were applied (WHO, 2017). Women were considered eligible to participate in the questionnaire if they were between 18 and 49 years with a live birth in the last year, and were excluded if they were not willing to participate or unable to understand the informed consent procedure. In total, based on sample size calculation, 740 women from the intervention site were sampled. They completed a 57-item questionnaire that included questions on demographics, knowledge and self-reported maternal health-seeking behavior. Interviewer administered paper and pen questionnaires were conducted in a private place at the selected households.

Surveys were also administered to CHWs during their monthly meetings in the intervention district of Sarwan. Of the seven meetings scheduled during the data collection period, three were randomly selected. All CHWs present at each of the three meetings were eligible to complete the CHW survey and none of them declined to participate. A total of 57 CHWs who attended these meetings completed the 14-question self-administered questionnaire. Research assistants that were trained to administer the interviews in Hindi assisted CHWs with lower literacy levels who indicated that they needed help.

#### Qualitative interviews

For the qualitative individual and group interviews, a subset of the quantitative participants was selected. Ten Panchayats (collection of villages) from the household surveys and CHW meetings were purposively selected. From these Panchayats respondents (CHW, women and men) who were available and willing to participate were selected. Inclusion and exclusion criteria for women and CHWs were the same as in the quantitative study. Semi-structured interviews were conducted among all respondent groups (CHWs, pregnant and lactating women and men). Themes covered ranged from experience with the MfM intervention, maternal health knowledge questions to other relevant contextual information (i.e. transportation). The qualitative interviews were conducted in a private place (i.e. house, backyard, Anganwadi center- basic healthcare provision facility in the villages and hospitals) and were recorded by audio device. Individual interviews lasted approximately 30–40 minutes and the group interviews (2–4 people) ranged between 60–90 minutes.

### Data analysis

#### Quantitative surveys

All completed surveys of women and CHWs were entered into Microsoft Excel in the field and analyzed in R [26]. Percentages were calculated for all categorical data. CHWs’ usage and perceptions regarding MfM were reported for the entire sample and also presented as a comparison between age groups (younger than or equal 30 vs older than 30) and educational level (completed lower secondary vs completed higher secondary) Data was stratified along the lines of age and education based on previous mHealth literature that has suggested possible differences in CHWs mHealth usages and perception based on age and education levels. Chi-square tests (fishers exact reported when one cell value was less than 5) were applied to test the difference usage and perceptions of MFM for (1) younger and older CHWS (2) CHWs who had completed lower secondary and those who had completed higher secondary.

#### Qualitative interviews

All individual and group interviews were recorded and transcribed verbatim, and translated from Hindi or Khortha (local language) to English. Data analysis was executed by means of content analysis in which a codebook was developed on the basis of the concepts from the conceptual framework and research questions (Pope, Ziebland, Mays, 2000). In the code scheme, each code was categorized under a corresponding theme and quotes from the participants were collected throughout the coding process. During the coding process, codes were reviewed. When necessary, codes were revised and new codes that emerged from the data were added. The codebook, coding and analysis was carried out by two researchers and checked on inter-coder agreement to enhance the study outcome’s validity. All coding and analysis were performed in MAXQDA version 12 (release 12.1.4).

## Results

In total 11 group interviews and 47 individual interviews were conducted. The main characteristics of participants are available in Tables 4 and 5. 740 household surveys were completed amongst women who received the intervention. The mean age of women who participated was 24 years (sd = 3.72), 35% of the women had not received any formal education (Table 4). The majority of CHWs were between 30–40 years old and had less than an 8^th^ grade education (Table 5).

**Table 4:** Socio-demographics of quantitative survey respondents.

*Variable*	*Women*
*N*	*740*
**Age**	
≤20	*185(25*.*1)*
≥21	*553(74*.*9)*
**Age at Marriage**	
≤18	*662(89*.*7)*
≥19	*76(10*.*3)*
**Religion**	
Hindu	*667(90*.*1)*
Others	*73(9*.*9)*
**Education**	
Illiterate	*259(35*.*0)*
Primary	*116(15*.*7)*
6th grade or higher	*365(49*.*3)*
**Husband’s Education**	
Illiterate	*174(23*.*5)*
Primary	*83(11*.*2)*
6th grade or higher	*483(65*.*3)*
**Caste**	
Scheduled Castes/Scheduled Tribes	*201(27*.*6)*
Other	*527 (72*.*4)*
**Occupation**	
Housewife	645(87.16)
Agriculture	77 (10.41)
Others	18 (2.43)

**Table 5:** Descriptive characteristics of community health workers.

Variables	N	%
***Age***		
≤29	132	36.4
30–40	191	52.6
≥41	40	11.0
***Education***		
Less than 8^th^ Grade	*245*	*58*.*8*
8^th^ Grade and Higher	*172*	*41*.*2*
***Caste***		
Scheduled Caste/Scheduled Tribe	*75*	*16*.*2*
Other Backward Caste	*251*	*54*.*1*
Others	*133*	*28*.*7*

### Facilitators and barriers to the CHWs’ acceptance and utilization of MfM

Through quantitative survey questions and qualitative interviews, factors that could serve as facilitators or barriers to the utilization of MfM were identified.

#### Technical support

The provision of support emerged as a facilitator for continued appropriate utilization of the mobile phone by CHWs. Support began with a two-day training but also included continuing support from the supervisors. CHWs received support in two main ways from the supervisor: (i) individually through home visits and phone calls, or (ii) as a group during cluster meetings. In the quantitative surveys 61% of CHWs indicated that they had received support in solving MfM related problems (Table 6). CHWs were asked to rank related services provided by NEEDS, those under the age of 30 were most satisfied with the training (32.4%) and the cluster meetings (32.4%), whereas CHWs older than 30 were most satisfied with the direct support from supervisors (48.0%) (Table 7). CHWs with only primary education were more satisfied with direct support (42.9%) (Table 8). There were no considerable differences among CHWs with secondary education or higher.

**Table 6:** Results of community health workers quantitative survey.

Variables	n	%
***Age***		
≤29	25	43.9
30–40	30	52.6
≥41	2	3.5
***Education***		
Lower than higher secondary	*29*	*50*.*9*
Higher Secondary or higher	*28*	*49*.*1*
***Caste***		
Schedule Caste/Scheduled Tribe	*n/a*	*n/a*
***START SAHIYYA***		
2007	50	87.7
2006	2	3.5
Other years	5	8.8
***START MfM***		
2011	4	7.0
2014	40	70.0
2015	13	22.8
***Usage MfM last month***		
0–2	31	54.4
3–4	20	35.1
5–6	3	5.3
> 6	3	5.3
***Utilized application components***		
Registration	56	98.2
ANC	47	82.5
INC	36	63.2
PNC	37	64.9
***Most utilized component***		
Registration	38	66.7
ANC	14	24.6
INC	2	3.5
PNC	2	3.5
***Difficult parts of application***		
Recharging balance	21	36.8
Submitting data	18	31.6
Charging	11	19.3
***Most difficult part of application***		
Submitting data and charging	*23*	*40*.*4*
***Easy parts of application***		
Understanding Hindi	48	84.2
Carrying the phone	47	82.5
Using the mobile phone	46	80.7
***Easiest part of application***		
Voice and Using the mobile phone	*25*	43.9
***Most satisfied***		
Understanding Hindi	33	57.9
Voice	25	43.9
Using the mobile phone	20	35.1
***Most satisfied NEEDS services***		
Training	15	26.3
Direct support from fieldworkers	21	36.8
Support during cluster meetings	20	35.1
***Support with application***		
No one	56	98.2
Family	43	75.4
Fieldworkers	35	61.4
***How MfM affected work***		
Made me more confident	46	80.7
Made my work more difficult	2	3.5
Helped me work better	54	94.7
Increased my workload	5	8.8
Had no effect	2	3.5
***Motivation***		
Respect from women/villagers	46	80.7
Made me more knowledgeable	39	68.4
Made me happier in my work	27	47.4
***How MfM affects health education***		
Educating women became easier for me	52	91.2
Educating women became more difficult for me	1	1.8
Had no effect	1	1.8
***How MfM affects relationships with women***		
Most women trust me and follow my instructions more	50	87.7
Most women trust me more, but still do not follow my instructions	4	7.0
Most women do not trust me and do not follow my instructions	1	1.8
Had no effect	2	3.5
***Noticed improvement in women’s health***		
Yes	51	89.5
No	3	5.3
***Satisfaction MfM***		
Yes	56	98.2
No	1	1.8
***Recommending MfM to others***		
Yes	54	94.7
No	2	3.5

**Table 7:** Demographic characteristics and outcomes of surveyed community health workers who utilized mobile for mothers [age group].

	≤30 n = 32	>30 n = 25	
	n (%)	n (%)	
***Usage MfM last month***			
0–2	*19(59*.*4)*	*12(48*.*0)*	*0*.*439*
3–4	*12(37*.*5)*	*8(32*.*0)*	
5–6	*0(0)*	*3(12*.*0)*	
> 6	*1(3*.*0)*	*2(8*.*0)*	
***Utilized application components (multiple answers)***			
Registration	*32(100*.*0)*	*24(96*.*0)*	0.027*
Antenatal care	*29(90*.*6)*	*18(72*.*0)*	
Intranatal care	*20(62*.*5)*	*16(64*.*0)*	
Postnatal care	*19(59*.*4)*	*18(72*.*0)*	
***Difficult parts of application (multiple answers)***			
Recharging balance	*11(34*.*4)*	*10(40*.*0)*	*0*.*366*
Submitting data	*12(37*.*5)*	*6(24*.*0)*	
Charging	*10(31*.*3)*	*6(24*.*0)*	
***Easy parts of application(multiple answers)***			
Understanding Hindi	*25(78*.*1)*	*23(92)*	0.018*
Carrying the phone	*25(78*.*1)*	*22(88*.*0)*	
Using the mobile phone	*28(87*.*5)*	*18(72*.*0)*	
***Most satisfied (max 3 answers)***	*N = 31*		
Understanding Hindi	*17(54*.*8)*	*16(64*.*0)*	*0*.*266*
Voice	*11(35*.*5)*	*14(56*.*0)*	
Using the mobile phone	*13(41*.*9)*	*7(28*.*0)*	
***Most satisfied NEEDS services (one answer)***			
Training	*11(35*.*5)*	*4(16*.*0)*	*0*.*251*
Direct support from fieldworkers	*9(29*.*0)*	*12(48*.*0)*	
Support during cluster meetings	*11(35*.*5)*	*9(36*.*0)*	
***How MfM affected work (two answers)***			
Made me more confident	*24(75*.*0)*	*22(88*.*0)*	*0*.*191*^*a*^
Made my work more difficult	*2(6*.*0)*	*0(0*.*0)*	
Helped me work better	*29(90*.*6)*	*25(100*.*0)*	
Increased my workload	*4(12*.*9)*	*1(4*.*0)*	
Had no effect	*2(6*.*0)*	*0(0*.*0)*	
***Motivation (3 answers)***			
Respect from women/villagers	*27(87*.*1)*	*19(76*.*0)*	*0*.*718*
Made me more knowledgeable	*21(67*.*7)*	*18(72*.*0)*	
Made me happier in my work	*16(51*.*6)*	*11(44*.*0)*	
***How MfM affects health education (one answer)***	***Three missing (999)***		*0*.*211*
Education women became easier for me	*28(96*.*6)*	*24(96*.*0)*	
Education women became more difficult for me	*1(3*.*0)*	*0(0*.*0)*	
Had no effect	*0(0*.*0)*	*1(4*.*0)*	
***How MfM affects relationships with women (one answer)***	***Two missing (999)***		*0*.*092*
Most women trust me and follow my instructions more	*26(90*.*7)*	*24(96*.*0)*	
Most women trust me more, but still do not follow my instructions	*0(0*.*0)*	*0(0*.*0)*	
Most women do not trust me and do not follow my instructions	*4(13*.*3)*	*0(0*.*0)*	
Had no effect	*0(0*.*0)*	*1(4*.*0)*	

* statistically significant

^a^ Fisher exact test was used.

**Table 8:** Demographic characteristics and outcomes of surveyed community health workers who utilized mobile for mothers [education group].

	Lower than Higher Secondary (n = *29)*	Higher Secondary or Higher (n = 28)	p
	n (%)	n (%)	
***Usage MfM last month***			
0–2	*16(55*.*2)*	*15(53*.*6)*	*0*.*037*
3–4	*7(24*.*1)*	*13(46*.*4)*	
5–6	*3(10*.*3)*	*0(0*.*0)*	
> 6	*3(10*.*3)*	*0(0*.*0)*	
***Utilized application components (multiple answers)***			
Registration	*29(100*.*0)*	*27(96*.*4)*	*0*.*237*^*a*^
ANC	*24(82*.*8)*	*23(82*.*1)*	
INC	*15(51*.*7)*	*21(75*.*0)*	
PNC	*15(51*.*7)*	*22(78*.*6)*	
***Difficult parts of application (multiple answers)***			
Recharging balance	*10(34*.*5)*	*11(39*.*3)*	*0*.*398*
Submitting data	*10(34*.*5)*	*8(28*.*6)*	
Charging	*11(37*.*9)*	*5(17*.*9)*	
***Easy parts of application(multiple answers)***			
Understanding Hindi	*20(69*.*0)*	*26(92*.*9)*	*0*.*083*
Carrying the phone	*20(69*.*0)*	*27(96*.*4)*	
Using the mobile phone	*24(82*.*8)*	*24(85*.*7)*	
***Most satisfied (max 3 answers)***			
Understanding Hindi	*17(60*.*7)*	*16(57*.*1)*	*0*.*061*
Voice	*13(46*.*4)*	*12(42*.*9)*	
Using the mobile phone	*5(17*.*9)*	*15(53*.*6)*	
***Most satisfied NEEDS services (one answer)***			
Training	*5(17*.*9)*	*10(35*.*7)*	*0*.*351*
Direct support from fieldworkers	*12(42*.*9)*	*9(32*.*1)*	
Support during cluster meetings	*11(39*.*3)*	*9(32*.*1)*	
***How MfM affected work (two answers)***			
Made me more confident	*20(69*.*0)*	*26(92*.*9)*	*0*.*062*^*a*^
Made my work more difficult	*2(7*.*14)*	*0(0*.*0)*	
Helped me work better	*27(96*.*4)*	*27(96*.*4)*	
Increased my workload	*4(14*.*3)*	*1(3*.*6)*	
Had no effect	*2(7*.*1)*	*0(0*.*0)*	
***Motivation (3 answers)***			
Respect from women/villagers	*24(85*.*7)*	*22(78*.*6)*	0.017*
Made me more knowledgeable	*15(53*.*6)*	*24(85*.*7)*	
Made me happier in my work	*8(28*.*6)*	*19(67*.*9)*	
***How MfM affects health education (one answer)***			
Educating women became easier for me	*24(92*.*3)*	*28(100*.*0)*	*0*.*11*
Educating women became more difficult forme	*1(3*.*8)*	*0(0*.*0)*	
Had no effect	*1(3*.*8)*	*0(0*.*0)*	
***How MfM affects relationships with women (one answer)***			
Most women trust me and follow my instructions more	*24(88*.*9)*	*26(92*.*9)*	*0*.*453*
Most women trust me more, but still do not follow my instructions	*0(0*.*0)*	*0(0*.*0)*	
Most women do not trust me and do not follow my instructions	*2(18*.*5)*	*2(7*.*1)*	
Had no effect	*1(3*.*7)*	*0(0*.*0)*	

* statistically significant

^a^ Fisher exact test was used.

Although the qualitative interviews did not provide reasons for these differences based on age and education, overall CHWs expressed satisfaction with support received. They were enabled in performing their duties by the help of supervisors provided in their use of the mobile application and the overall functioning of the mobile.

*“He [the supervisor] goes to their home*, *and asks them to sit on the chair and then he tells them to type to the people*. *He tells us [CHWs] to type like you do in front of me*. *Whatever you have a problem I am here for it*. *You do the typing”*(CHW 2, Group Discussion 1)

#### MFM technology

In discussion about challenges with MfM technology, language was frequently mentioned. CHWs older than 30 found it easier to understand the Hindi utilized in the MfM application compared to those younger than 30 (Table 7). However, younger CHWs found using the mobile phone in general to be easier than older CHWs. There were also differences between educational levels and more educated CHWs found it easier to understand Hindi (92.9%) compared to CHWs with less education (69.0%) (Table 8). There were no reported differences in ease of using the mobile phone based on education level. Younger CHWs (younger than 30) reported more problems than the older CHWs with submitting data (37.5 vs 24.0) and charging phones (31.3% vs 24.0%). We also found statistically significant differences in what components the CHWs found easiest based on their age group (p = 0.02).

In qualitative interviews with CHWs all groups hypothesized that typing in English would be harder for CHWs who had not received any formal education. However, the group of CHWs with the highest average level of education (11th grade) in general expressed the most reservations about typing in English. The youngest respondents expressed the strongest positive feelings towards typing in English, but this was also the group with the second lowest average education level. Some CHWs shared that they experienced problems with the mobile phone itself and the cellular network. Network and internet service disruptions were system level challenges, which affected the CHWs ability to submit collected data. However, there was some indication that problems with the mobile phone could stem from technical faults of the phone or simply the difficulty related to using unfamiliar technology. The CHW who admitted to having problems with MfM had the lowest education level recorded–grade 5.

#### CHWs’ perceptions of MfM’s influence on knowledge and performance

CHWs and other stakeholders discussed their views on the effects of MfM in terms of knowledge and performance, both of CHWs and women.

#### Change in knowledge

Most of the CHWs perceived that the mobile phone was related to a change in knowledge amongst the women but equally amongst themselves. In the survey, 68.4% indicated that MfM had made them more knowledgeable on recommended maternal health practices. CHWs also discussed the change in knowledge recall in the mothers they served.

*“Yes*, *there is a big difference*. *Before they do not keep in mind what I told them but with the use of the mobile they remember*. *First we do registration and then ANC*, *and after completion of these five processes*, *they keep everything in mind*.*”* (CHW 1, Group Discussion 2)

#### Change in CHW performance

94.7% of CHWs in the survey indicated that MfM helped them work better and 80.7% reported increased confidence. A few CHWs (6%) reported that MfM made their work harder; they were all in the younger age bracket. 7.14% of CHWs in the lower education group reported that MfM made their work more difficult (Table 8).

In the qualitative interviews, CHWs frequently reported an overall change in their performance. CHWs shared that the mobile phone improved their ability to perform tasks by enlightening them on issues which they were previously ignorant about, such as the number of required ANC visits. They explained that the intervention also improved their ability to explain concepts (such as the need for STI testing) to their community members. MfM also assisted with their recall and delivery of essential information. Prior to the implementation of MfM, they had books to assist with their recall, however, the mobile application appeared to improve their ability to deliver consistent information to all women. In this way MfM reduced their mental workload but also improved efficiency and the accuracy of information relayed.

“*We were not able to remember all the things*. *Since when we got the mobile we do not leave anything out and are able to give all the information to the pregnant women*. *We all always left many things out (before the mobile) and by this (the mobile) nothing is left out*.” (CHW 4, Group Discussion 2)

Auxiliary Nurse Midwife (ANMs) interviewed also recognized the benefits of MfM for CHWs. ANMs reported that they perceived that MfM increased CHWs’ confidence, improved case-management, improved CHWs’ performance (i.e. number of visits and awareness) and made the CHWs’ work easier. One ANM highlighted that MfM could serve as a tool for monitoring and evaluating the CHW’s work. The local government officer mentioned that he received positive reactions from the CHWs about the program, such that community members were now more interested in listening to the information CHWs presented.

### Contextual factors influencing maternal health-seeking behavior

Context was broadly defined and we explored factors such as the relational dynamics or relationships between CHW and their communities, as well as known facilitators and barriers to maternal health seeking behavior such as barriers related to transportation or lack of autonomy in decision-making.

#### Relationships between CHWs and women

Surprisingly CHWs and women appeared to have different interpretations of their relationship and the influence of mHealth on it. In the quantitative surveys, 80.7% of CHWs indicated that they gained respect from women and community members because of MfM utilization. 87.7% also indicated that MfM had led to an increase in the village women trusting them and following their directions. However, 13.3% of CHWs younger than 30 reported that women did not trust them or follow their instructions. Of these CHW 18.5% had lower education level and 7.1% had secondary education or higher (Table 8). None of the older CHWs reported problems with distrust of community members.

In qualitative interviews, CHWs generally expressed that they felt their work and advice were taken more seriously with the introduction of the mobile intervention.

*“When we were telling on the paper then the pregnant women said that*: *look*, *sister is telling something*, *they were paying less attention*. *But when the mobile is telling then she is also thinking that there must be something in this*. *The Mobile is asking me (the mother)*. *Sister (CHW) is not asking us*. *Therefore they give more attention to the mobile*.*”* (CHW 3, Group interview 2)

*“I am not telling*. *The mobile is telling*. *They will hear its words*. *Whoever is nearby they also become silent and will here all things*.*”* (CHW 1, Group interview 4)

CHWs however made a distinction between the womens’ trust of them as individuals and their trust of the information they provided. They frequently gave examples of women trusting them enough to share sensitive information such as a first suspicion of pregnancy or the presence of infections that they were yet to discuss with their in-laws or family members. As indicated above, the CHWs felt that the mobile phone increased the credibility of their advice. In contrast, the women did not make similar distinctions between their trust of the CHW as a person and increased acceptance of her advice now she had a mobile phone. As five of women explained the CHW was a known figure to them, she had already been working amongst them and as such there was no reason to doubt her.

*“Like whatever information we are getting through the mobile it is good and like whatever sister is telling that also is good*.*”* (Mother 11)

Men corroborated that the CHW was trusted within their respective communities and had a significant role. Trust in the CHW was discussed in one third of the interviews with men. Specific instances of this included discussing that in the absence of the husband or in-laws, CHWs could play a role in decision-making.

*“The CHW play a very important role in maternal health*. *Whenever someone needs her even in the night*, *she comes with her to the clinic*. *If her husband is outside of town than there are many people in the family like her in-laws or other older members of family*, *anyone can decide*. *And there is also (the) CHW”*. (Man 15)

Two of the women shared that distrust could be a reason why women did not follow CHW’s advice. They did not express having a distrust of the CHW themselves, but indicated that other women may have such issues. These women who had high education attainment themselves attributed the distrust to education level of the clients (pregnant or lactating women) mostly.

#### Autonomy/Decision Making/Familial Influence

In the quantitative survey, 26% of women indicated that the decision to seek antenatal care was theirs but for more than half of the sample their husbands made the decision. In-laws made only 6% of decisions regarding antenatal care attendance (Table 9). A similar pattern was noticed when women were asked who decided on the delivery location. 43% of women indicated that the decision to deliver at a hospital was taken by their husbands. For 29% of women their in-laws took the decision and only 17% of women answered that they made the decision themselves.

**Table 9:** Maternal health seeking decisions and behaviors.

*Variable*	*Post-Intervention*
N	*740*
**ANC Decision**	
Respondent	*188(26*.*1)*
Husband	*477(66*.*2)*
In-laws	*44(6*.*1)*
Others	*31(1*.*6)*
**Delivery at Health Facility**	
Yes	*125(23*.*2)*
No	*542(76*.*8)*
**Decision to Deliver at Health Facility**	
Woman	*29(1*.*75)*
Husband	*72(43*.*4)*
In-laws	
**Reason for not delivering at a health facility**	
Not necessary/Not customary	*31(18*.*9)*
Cost too much	*5(3*.*0)*
Too far/No transport	*12(7*.*3)*
No confidence in available personnel	*74(45*.*1)*

The qualitative information showed a difference in perceptions about decision makers. CHWs attributed decision-making more frequently to guardians (father-in-law and mother-in-law), with greater mention of mother-in-laws. Two of the CHWs also expressed this acknowledgment of the role of guardians in their view of their duties as not only to educate the pregnant woman but also her guardians and husband. Majority of the CHWs interviewed discussed the role of the mother-in-law as extending to other health behaviors beyond ANC attendance and delivery at a health facility. Mother-in-laws were also frequently cited as the authority in the homes and thus the reason women were unable to comply with other recommended maternal health practices, such as avoiding lifting heavy objects and getting adequate rest. Eight CHWs when asked to cite reasons why a woman might have her baby at home despite receiving information about the importance of facility based delivery, highlighted the role of guardians. Women also discussed the role of guardians in their ability to follow instructions of healthcare workers and selecting a healthcare location decision of delivery location. One woman presented the decision making power of the guardians not as controlling or negative but as a form of care.

*“The decision is taken by the guardian*. *The pregnant woman is the problem [experiencing the labor] so the decision is taken by the guardian*. *If the guardian wants to take us there then we will go*. *And if they want the delivery to take place at home then it’s their will*.*”* (Mother 18)

Two women in interviews asserted that they and sometimes their husbands determined health-seeking behavior. Men, on the other hand, frequently indicated that their wife could take decisions herself, but also cited the guardian as responsible for decision-making.

#### Financial access

Financial concerns were frequently mentioned as barriers to care. While some women mentioned the JSK scheme only one woman actually mentioned receiving the promised amount. The CHWs shared that the change in the JSK payment scheme had led to problems in the incentive scheme working optimally. CHWs also reported that from their experience women and their families were discouraged by the costs of hospital stay.

#### Household responsibilities and other non-financial issues

Another barrier to care discussed by CHWs and women in qualitative interviews, was the workload at home. In 15 interviews it was mentioned that women might be unable to seek healthcare, such as attending ANC visits, because of households’ chores and responsibilities. Having young children in the home was also often cited as a barrier to both receiving antenatal care and delivering at a health institution.

*“Now its 72 hours and they have to live (continue with daily activities)*. *So there are many people who cannot live for that many days*. *They do not see that they are getting the facility*. *My child will be healthy…These things they are not thinking*. *They understand that if I will remain there for 72 hours then who will fence my goats that are in my house*. *And there are two more children*. *Will I take both of the children to the hospital then who will remain in the house*. *Who will go to the hospital? So these kinds of problems exist*.*”* (CHW 3, Group Discussion 1)

#### Transportation issues

The government program, Mamta Vahan scheme, received both praise and criticism from women and CHWs alike. The scheme was described as meeting a need for transportation that all of the communities appeared to share. Some women and CHWs reported positive experiences with the scheme. However, there were also complaints about the late arrival of the van as a hindrance to reach the health facilities in a timely manner for delivery. A mother describes she had her first baby at home because of the vans’ delay.

*“(The) Van came and went back*. *Baby was already born when it came…Sahhiya called the van (mamta vahan) but I had already delivered when the van had half reached*.*”* (Mother 8)

However, some of the CHWs reported that women frequently delayed alerting their families about the onset of labor. Therefore, by the time the van was called for and arrived labor was too advanced for women to be moved. Some CHWs shared that women could be taken to the health center by an auto rickshaw so the Mamta Vahan van’s late arrival was not the biggest problem.

Men also mentioned the Mamta Vahan as they were discussing their wife’s delivery. Two men who pointed out the limitations of the Mamta Vahan, such that it only goes to one predetermined hospital, and that the CHW is limited to only call one ambulance and not others. Both are probably related to the Mamta Vahan being utilized in combination with the government hospital solely.

*“I took my wife to hospital for delivery because there is better solution of treatment*. *We get every treatment ready there*. *Money is the influential factor because doctors sometimes refer patients to the big cities for better cure*. *This is very expensive even transportation cost*. *Mamta Vahan is free but we cannot use Mamta Vahan to take them to other cities*.” (Man 2, Group Discussion 1)

#### Availability of services

Two factors emerged that were related to the availability of services. One of these, mobility of women, was directly related to MfM outcomes, while the second was related to access to health services.

In the qualitative interviews “mobility” emerged as a possible reason for women failing to attend complete ANC or have completed ANC, delivery or PNC records in the MfM system. It was reported that some women moved in order to find employment; this form of mobility was only mentioned in one panchayat. However, mobility generally arose from women moving from their marital homes back to their parents’ home in their 2^nd^ or 3^rd^ trimesters. This interrupted the continuum of care, as women did not complete their ANC or receive PNC care in the same health center.

*“Like she lived here for six months then she went to her mother’s house after some days*. *So I was not able to do her ANC here*. *So this can be the reason*.*”* (CHW 1, Group Discussion 3)

Availability of services at local health facilities and the distance to better-equipped facilities also played a role. In one of the communities where group discussions took place the CHWs noted that women were only able to have their first and second ANC at the local health facility but then would have to go to the PHC for further tests. This discouraged women from attending later ANC visits.

## Discussion

Program evaluations of mhealth applications have focused on improving CHW performance and expected health outcomes. However, these program evaluations rarely go deeper into the factors explaining success or failure. While some studies have explored CHWs’ and clients’ (pregnant women or new mothers) perceptions of mHealth interventions, we did not identify any studies that have attempted to explore factors that explain an effective CHW-utilized intervention from multiple perspectives in rural India. This study aimed to understand which factors were related to the observed outcomes of a CHW-utilized mHealth intervention (Table 10 outlines 6 key messages). MfM program designers clearly outlined expected outcomes, including improved maternal health-seeking behavior in pregnancy, and the intermediary outcomes: such as improved knowledge among community health workers and pregnant women. However, the intervention did not explicitly address broader contextual factors, which could contribute to MfM outcomes. In this paper we have highlighted the importance of contextual factors in the development of a Theory of Change for the design of future mHealth interventions.”

**Table 10:** Key messages.

Key Messages • MfM improves CHWs’ perceptions of confidence and performance of maternal health related tasks by providing a systematic source for information delivery. • CHWs’ experiences of training, support and utilization of mobile health technology are influenced by their age and education levels. • mHealth interventions are limited in their impact on maternal health decision seeking as their effects are mediated by: • Contextual factors such as the women’s responsibilities for the home, the need to arrange childcare during clinic visits or delivery and the positioning of men and mother-in-laws as key decision makers. • Issues related to absence of adequate infrastructure related to transportation in rural areas proved to be a significant barrier in accessing care. • In addition, it is essential that program developers consider contextual factors, such as those raised in our study, in the design of theory of change models as these could potentially influence outcomes and the achievement of mHealth intervention goals.

In a context where women have limited or no prior exposure to mobile phone applications the appropriate training and support needs vary by age and education level. In the Indian context, younger CHWs preferred group training and learning methods, whereas older CHWs seemed to prefer the one-on-one attention. Women with higher education levels also preferred the direct attention from supervisors to the group conversations. Reasons for this difference did not arise in the qualitative interviews, but some assumptions can be made from research on learning. Although we did not find research specific to the Indian context, Cekeada’s (2012) work on multi-generational workplaces suggests that there are differences in learning styles correlated with different generations [27]. However, the literature on the effect of age on mHealth utilization of CHWs remains mixed; with some studies citing youth as an advantage and others as a limitation [28,29]. It is important to note that there were only 3 indications of statistically significant differences between CHWs of different age groups and education levels. However, considering the small survey sample size and taking into account the information provided in the qualitative interviews, we believe that this is an important factor to explore further.

In a context where CHWs have sufficient knowledge to utilize the mHealth application effectively, without health system-related barriers, the intervention could lead to CHWs reporting increased knowledge and confidence. CHWs reported that utilizing MfM improved their maternal health knowledge. In general they also reported that the use of MfM improved their performance by making them more confident in the information they shared. This finding is supported by other mHealth studies, which have shown that mobile phones and mobile applications are able to increase confidence amongst CHWs [8]. The common mechanism related to improved confidence and performance was the functioning of MfM as a job aid. MfM provided CHWs with accurate information constantly and in a systematic manner. This reduced their mental workload, as they were no longer responsible for remembering what questions needed to be asked and which information had to be conveyed.

In a context where women have limited decision-making power and face multiple challenges in access to care, including financial and socio-cultural barriers, and challenges related to health system constraints, mHealth is evidently limited in its ability to address these constraints.

When an mHealth intervention is implemented within an existing intervention, such as a CHW program, factors linked to program implementation can affect the intervention outcomes. The ASHA system in India, a national government-led program delivered at the state level has many positive outcomes, but there are also challenges within the programs. The ASHA’s nomination within the community is sometimes the result of political connections. There were some issues with trust reported regarding certain CHWs, linked to general issues of trust between CHWs and communities [19].

In a context where decisions on maternal health-seeking are characterized by unequal power relations in the household, mHealth interventions that target only women may not lead to expected outcomes. MfM messages were tailored and delivered to pregnant or post-natal women. While husbands and in-laws could listen to the message, they were not actively targeted. Both quantitative and qualitative indicated that that maternal health decisions were frequently not made by women. Over half of the women surveyed indicated that someone else made the decision of delivery location. This is similar to findings on decision making in other LMICs [30]. It is important to note that while quantitative results suggested that men were more likely to make decisions about delivery locations, qualitative results suggest that (mother-)in-laws influence a woman’s ability to uptake ANC services [31].

### Strengths and limitations

The findings from this study highlight key factors that could influence the intended outcomes of the mHealth intervention. The triangulation of data sources and methods present an in-depth understanding of the complexity of mHealth interventions and the context in which they operate.

A limitation of this study was that the primary researcher and other research assistants were not local to the research environment. Attempts were made to limit this by engaging a local translator and involving local experts in instrument design and data interpretation. In addition, simple expressions in Hindi and Kortha can have nuanced meanings, which are difficult to capture in English. To address this, interviews and interpreters had daily reflections meetings. Also qualitative and qualitative data collection occurred concurrently, so while qualitative interviews provide additional explanations for a number of quantitative results, some points were not explored in the qualitative interviews.

## Conclusions

The Theory of Change proposed by the program developers provides a guide, which is useful for intervention development. However, we propose that contextual factors must be explicitly addressed in the design of mhealth interventions. Program developers and designers need to explore contextual and implementation factors. A failure to do this could lead to an underestimation or overestimation of mHealth effectiveness.

## Supporting information

S1 File CHW survey. (PDF)

S2 File Household survey. (PDF)

S3 File Qualitative guide. (PDF)
