# Supplementary material for: “I am not telling. The mobile is telling”: Factors influencing the outcomes of a community health worker mHealth intervention in India
Source: PLoS One. 2018 Mar 27;13(3):e0194927. doi: 10.1371/journal.pone.0194927 (PMC5870994; doi:10.1371/journal.pone.0194927)
Supplement: S1 File — (PDF) [file pone.0194927.s001.pdf]

S1 File. CHW Survey

**INSTRUCTION: Write or circle your answer(s) to the questions below.**					
**Age**					
**Education level**	**Primary**	**Secondary**	**Higher Secondary**	**College/University**	
**When did you become a Sahiyya?**	**2005**	**2006**	**2007**	**2008**	**2009**
	**2010**	**2011**	**2012**	**2013**	**2014**
**When did you join MfM?**	**2011**	**2012**	**2014**	**2015**	

**1.**	In general, how often did you use MfM in the last month?	**0-2**	**3-4**	**5-6**	**More than 6**
**2.**	Which components of the MfM application do you use during work? (Circle all that apply)	**Registration**	**ANC**	**INC**	**PNC**
	Of the components you chose above, which do you find most useful?				
**3.**	Which parts of the MfM application were difficult? (Circle all that apply)	**Voice**	**Pictures**	**Size of picture**	**Understanding Hindi**
		**Words/Text used**	**Size of words/text**	**Using the mobile phone**	**Recharging money to mobile phone**
		**Charging the battery**	**Carrying the phone**	**Typing on the phone**	**Submitting the data**
	Of the parts you chose above, which do you find the most difficult?				
**4.**	Which parts of the MfM application were easy? (Circle all that apply)	**Voice**	**Pictures**	**Size of picture**	**Understanding Hindi**
		**Words/Text used**	**Size of words/text**	**Using the mobile phone**	**Recharging money to mobile phone**
		**Charging the battery**	**Carrying the phone**	**Typing on the phone**	**Submitting the data**
	Of the parts you chose above, which do you find the easiest?				

**5.**	Which parts of the MfM application are you most satisfied with?<br><br>(Circle a maximum of 3 answers)	**Voice**	**Pictures**	**Size of picture**	**Understanding Hindi**	
		**Words/Text used**	**Size of words/text**	**Using the mobile phone**	**Recharging money to mobile phone**	
		**Charging the battery**	**Carrying the phone**	**Typing on the phone**	**Submitting the data**	
**6.**	Of which services related to MfM offered by NEEDS are you most satisfied with?	**Training**	**Monitoring**	**Direct support from fieldworkers**	**Support during cluster meetings**	
**7.**	Who helps you with the MfM application: It can be related to entering data, recharging money on the phone, saving data or submitting the data? (Circle all that apply)	**Field Workers**	**ANM**	**Other Sahiyyas**		
		**Family**	**Friends**	**No one**		
**8.**	How did MfM affect your work as a Sahiyya? (Circle a maximum of 2 answers)	**Made me more confident**	**Made my work more difficult**	**Helped me work better**	**Increased my workload**	**Had no effect**
**9.**	What motivates you most in continuously using MfM? (Circle a maximum of 3 answers)	**Made my work more efficient**	**Made me happier in my work**	**Made me more knowledgeable**		
		**Increased incentives**	**Respect from women/villagers**	**Respect from ANM**	**I don't use MfM continuously**	
**10.**	How did MfM affect the way you educate women about maternal health in your village?	**Educating women became easier for me**	**Educating women became more difficult for me**	**Had no effect**		
**11.**	How did MfM affect your relationship with mothers and pregnant women in your village?	**Most women trust me and follow my instructions more**	**Most women trust me more, but still do not follow my instructions**			
		**Most women do not trust me and do not follow my instructions**	**Had no effect**			
**12.**	If you compare the current situation with the previous situation of maternal health before MfM, have you noticed any improvement in the health of mothers and pregnant women in your village?	**Yes**	**No**			
**13.**	In general, are you satisfied with using MfM?	**Yes**	**No**			
**14.**	Would you recommend MfM to other Sahiyyas who still do not have MfM?	**Yes**	**No**			
