# Supplementary material for: “I am not telling. The mobile is telling”: Factors influencing the outcomes of a community health worker mHealth intervention in India
Source: PLoS One. 2018 Mar 27;13(3):e0194927. doi: 10.1371/journal.pone.0194927 (PMC5870994; doi:10.1371/journal.pone.0194927)
Supplement: S2 File — (PDF) [file pone.0194927.s002.pdf]

For Research Purpose only

ID Code ☐☐☐☐☐

Baseline Survey on

"Mobile for Mother"

Household Questionnaire

Baseline HH questionnaire

CONSENT FORM

Namaste. My name is ____ and I am working with NEEDS. We are conducting a survey about the health of women and use of health facilities. We would very much appreciate the participation of your household in this survey.
I would like to ask you some questions about your household. The survey usually takes about 20 minutes to complete. Whatever information you provide will be kept strictly confidential. Participation in this survey is voluntary and you can choose not to answer any question or all of the questions. However, we hope that you will participate in this survey since your participation is important. At this time, do you want to ask me anything about the survey?

ANSWER ANY QUESTIONS AND ADDRESS RESPONDENT'S CONCERNS.

In case you need more information about the survey, you may contact Mr. Murari Chowdhary. (GIVE CARD WITH CONTACT INFORMATION)

May I begin the interview now? Yes----1 No------2

Signature of interviewer:

Date:

Consent YES / NO (Please circle)

RESPONDENT AGREES FOR INTERVIEW...........1 **RESPONDENT DOES NOT AGREE FOR INTERVIEW....................2——> END**

**Section . Identification Information**

101.	Name of the Respondent<br>mÙkjnkrk dk uke	
102.	Household Address<br>**?kj dk irk**	________________No. □□□
103.	Village Name **xzke**	________________No. □□
104.	Main village or hamlet<br>eq[; xzke ;k Vksyk	Main village………………………1<br>Hamlet…………………………….2<br>(Name……………………………..)
105	How many months ago was your last delivery?	No. □□
106.	Sub-centre Name **miLokLF; dsUnz**	__________________ No. □□

107. Interviewer Signature<br>lk{kkRdkj drkZ	---	108. Date<br>□□/□□/□□□□
109. Supervisors signature<br>i;Zos{kd	---	110. Date<br>□□/□□/□□□□

111. Result Code	lk{kkRdkj dk ifj.kke	□
112. Total No. of visits		□

(Result codes)
1. Completed; 2. Completed after revisit, 3. Refused; 4. Respondent/Entire household Absent; 5. Incomplete; 6.Other -------------------- (Specify)

201.	Since how long you are living in this area?<br>**vki ;gk¡ dc ls jg jgs gS **	---------------- Years
202.	**vkidk /keZ D;k gS **<br>What is the your religion?	Hindu ………………………….1<br>Muslim …………………………2<br>Christian ....………………….3<br>Sikhism……………………....4<br>Sarna ....……………………5<br>Others (specify) ....………..9
203.	**D;k vki vuqlwfpr tkfr] tutkfr ;k vU; fiNM+h tkfr ds gSa ;k buesa ls dksbZ ugha gSA**<br>Does the respondent belongs to scheduled caste, scheduled tribe, OBC or none of them?	SC ………………………………1<br>ST ………………………………2<br>OBC ...……………………………3<br>Others (Specify) ....……….9
204.	**vkius dkSu lk mPpre ntkZ ikl fd;k gS **<br>Which is the last class/ standard you attended?<br>(Record exact class)	Grade ………………….....□□
205.	**vkids ifr us dkSu lk mPpre ntkZ ikl fd;k gS **<br>Which is the last class/ standard your spouse attended? (Record exact class)	Grade ……………………..□□
206	**vkidk eq[; O;olk; D;k gS **<br>What is your primary occupation?<br>**mi;qDr dksM ns[kdj Hkjasas**	______________________ □
207	**vkids ifr dk eq[; O;olk; D;k gS **<br>What is the primary occupation of your husband?<br>**mi;qDr dksM ns[kdj Hkjasas**	______________________ □
208.	**vki vius fiNys tUefnu ij fdrus o"kZ ds Fksa **<br>How old were you at the time of your last birthday? (Record in years)	□□
209.	**'kknh ds le; vkidh mez D;k Fkh **<br>What was your age at the time of your (first) marriage? (Record in years)	□□
210.	**igys xHkZ/kkj.k ds le; vkidh mez D;k Fkh **<br>What was your age at the time of your (first) pregnancy? (Record in years)	□□
211.	**vafre xHkZ/kkj.k djus ds le; vkidh mez D;k Fkh **<br>What was your age at the time of your last pregnancy? (Record in years)	□□

212. Have you ever heared about the following: (Knowledge): **D;k vkidks bu ckrksa ds ckjs esa tkudkjh gS **

Components		Source of information (multiple possible) (Use codes)

212 A. Do you know why you need to visit ANM/doctor during your pregnancy?	1 YES / 2 NO	If Yes, ask why?<br>[ ] Helps to identify (severe) problems and provide solutions<br>[ ] It will minimize risks<br>[ ] Receive additional services (TT, iron)<br>[ ] Other (correct answers)<br>[ ] No correct answer<br>(respondent needs to give spontaneous answers
212 B. Do you know why you need to take TT injection?	1 YES / 2 NO	If yes, ask why<br>[ ] It will protect the baby from tetanus<br>[ ] No correct answer
212 C. Do you know why you need to IFA (iron) tables	1 YES / 2 NO	If yes, ask why<br>[ ] It increases blood and will protect fron anemia<br>[ ] No correct answer
212 D. Do you know the danger signs in pregnancy?	1 YES / 2 NO	If Yes, mention all<br>[ ] Bleeding from vagina during pregnancy<br>[ ] Severe abdominal pain during pregnancy<br>[ ] Severe headache with blurring of vision<br>[ ] Convulsions / loss o Consciousness<br>[ ] No correct answer<br>mention

212 E. Do you know the danger signs during labour/ delivery?	1 YES / 2 NO	If Yes, mention all<br>☐ If the membranes/ water is broken/coming out before the expected date.<br>☐ It the women has convulsions<br>☐ Labour for more then 12 hours<br>☐ Placenta does not come out within 30 minutes after the baby is delivered<br>☐ No correct answer
212F Do you know why 5 cleans are important during delivery?	1 YES / 2 NO	If yes, ask why<br>☐ It limites infection (and death) both in baby an mother<br>☐ No correct answer
212 G. ANC care: Ikzloiqo Z ns[kHkky<br>1. Antenatal checkups are important<br>IkzloiqoZ tk¡p egRoiq.kZ gS A<br>2. Iron folic tablets<br>vk;j.k dh xksfy;k¡<br>3. TT injection<br>Vh Vh dh lqbZ<br>4. Danger sign of pregnancy<br>Ikzlo ds nkSjku [krjs ds ladsr		Source<br>☐<br>☐<br>☐<br>☐
212 H. Delivery care<br>1. Five Cleans<br>Ik¡kp lQkbZ	1 / 2	☐
212 I. RTI/STI	1 / 2	☐
212 J. HIV/AIDS	1 / 2	☐

Codes for source of information: Any health professional-1 ; Newspaper/Magazine-2, Television-3, Radio-4; NGO worker-5; Husband-6. Sahiyya- 7; Friends-8; Other- 9

Baseline HH questionnaire

	Section. Reproduction (Pregnancy History):	

301. Information about obstetric history (first pregnancy first)

Pregnancy	Alive (A)/ Abortion or Still birth (AS)/ Died (D)	If 'AS' If not alive during pregnancy, after how many months did the pregnancy end?	If born alive but not alive now, when did the baby die? (1) < 6 weeks (2) >6 weeks but < 1 year (3) > 1 year	Female	Male
1					
2					
3					
4					
5					
6					
7					
8					

302	If given a choice, how many children would you like to have in your life?<br>**vxj vkidh bPNk ij fuHkZj gks rks vki fdrus cPps pkgsaxha **	Number _______ ☐<br>Other--(Specify)

	**Now I want to talk about your most recent pregnancy (that ended in a live birth within the last 12 months.**	
401	**Was this pregnancy planned?**	Yes ....1<br>No 2
402.	**(Ukke) ------------------------------ds le; tc vki xHkZorh Fkha rc Dk vki izloiwoZ tkap ds fy, xbZ Fkh **<br>When you were pregnant with your youngest child, did you go for an antenatal check-up?	Yes ....1<br>No ....2<br>(skip to 407)
403.	**vkius fdldks fn[kk;k \\ fdlh vkSj dks **<br>Whom did you see at the time of first antenatal check up?	Doctor ....1<br>ANM ....2<br>Nurse/Midwife/ LHV ....3<br>Other health professional ....4<br>Traditional birth attendant (DAI) ....5<br>Sahiyya........6<br>Other …………(specify) 9
404.	**vkius fdlh vkSj dks fn[kk;k D;k \\ (iwjh xHkkZoLFkk ds nkSjku**<br>Who else did you see during the entire period of pregnancy?<br>Anyone else?<br>Record all persons seen.	Doctor ....A<br>ANM ....B<br>Nurse/Midwife/ LHV ....C<br>Other health professional ....D<br>Traditional birth attendant (DAI) ....E<br>Sahiyya........F<br>No one........G

		Other …………(specify) ………………………………X
405.	**tc vki xHkZorh Fkh tc igyh ckj tk¡p ds fy, igyh ckj izloiwoZ ;s ns[kus xbZ Fkh fd lc Bhd&Bkd gS ;k ugha dksbZ leL;k Fkh **<br>When you were pregnant with your youngest child, did you go to antenatal care for the first time to check that everything was fine or because you had a problem?	Because of a problem ……………………………1<br>To check only ………………………………………2
406.	**izloiwoZ tk¡p dk fu.kZ; fdlus fy;k **<br>Who mainly made the decision to go for an antenatal check-up?	Respondent ……………………………………01<br>Husband ……………………………………02<br>In-Laws ……………………………………03<br>Parents ……………………………………04<br>Sister/Sister-In-Law ……………………………05<br>Other member in husband's family ………………06<br>Other member in respondent's family ……………07<br>Relatives ……………………………………08<br>Friend/Neighbour ………………………………09<br>TBA/Field Worker ………………………………10<br>Other …………(specify) ……………………………99
407.	**tc (Ukke) -------------------------------ds le; vki xHkZorh Fkh] rc D;k dksbZ LokLF; dk;ZdÙkkZ izloiwoZ tk¡p ds fy, vkids ?kj vk;k Fkk **<br>When you were pregnant with your youngest child, did any health worker visit you at home for an antenatal check-up with a physical examination?	Yes ……………………………………………………1<br>No ……………………………………………………2
408.	Check 402 and 407:	Yes in either ………………………………………………1<br>(skip to 410)<br>No in both ………………………………………………2
409.	**vkidh izloiwoZ tk¡p u gksus dk eq[; dkj.k D;k Fkk **<br>What is the main reason you did not receive an antenatal check-up?	Not necessary ………… 01<br>Not customary ………… 02<br>Cost too much ………… 03<br>Too far/no transportation ………… 04<br>Poor quality service ………… 05<br>No time to go ………… 06<br>Family did not allow ………… 07<br>Lack of knowledge ………… 08<br>No health worker visited ………… 09<br>Nobody to accompany ………… 10<br>Other …………(specify)……………………………99<br>→ **Any answer go to 417**
410.	**izlo iwoZ tk¡p ds le;] D;k vkidks dksbZ dkMZ feyk Fkk ** Did you receive any health card at the time of ANC check-up?	Yes ……………………………………………………1<br>No ……………………………………………………2<br>(skip to 413)
411.	**vxj gk¡] rks D;k eSa ns[k ldrh gw¡ **<br>If so, may I see it, please?	Yes, seen ……………………………………………1<br>Yes, not seen …………………………………………2<br>No card ……………………………………………3<br>(if 2 or 3, skip to 413)
412.	**dkMZ ns[kdj Hkjsa**<br>Enter the following data from the card	Total no. ANC visits: □<br>First visit □□/□□/□□□□<br>Last visit □□/□□/□□□□<br>IFA tablets (No.) □□□

		TT 1 □□/□□/□□□□<br>TT2 □□/□□/□□□□
413.	**bl xHkkZoLFkk ds nkSjku vkidh fdruh ckj izloiwoZ tk¡p dh xbZ **<br>How many times did you receive antenatal check-ups during this pregnancy?	TOTAL ANC Check ups ………….. □<br>Do you remember approximately when they took place?<br>Trimester No. of check ups<br>1st (0-3 months) ………………… □<br>2nd (4 –6 months) ……………………… □<br>3rd (7-9 months) ……………………… □
414.	**bl xHkkZoLFkk ds nkSjku D;k fdlh Hkh izloiwoZ tk¡p ds le; buds fy, de ls de ,d ckj tk¡p dh x;h Fkh ?**<br>Did you have the following performed at least once during any of your antenatal check-ups?	**Yes No**<br>Weight measured …………………....1 2<br>Height measured …………………....1 2<br>Blood pressure checked …………...1 2<br>Blood test ……………………………....1 2<br>Urine test ……………………………....1 2<br>Internal Exam ………………………...1 2<br>Abdomen examined ………………...1 2<br>X-Ray………………………………....1 2<br>Sonogram/Ultrasound. ………………..1 2
415.	**bl xHkkZoLFkk ds nkSjku de ls de fdlh ,d izloiwoZ tk¡p ds fy, D;k vkidks buesa ls fdlh Hkh fo"k; ij lykg feyh **<br>Did you receive advice on any of the following during at least one of your antenatal check-ups for this pregnancy from the ANM/ Sahhiyya?	**YES NO**<br>Diet …………………………………....1 2<br>Danger signs of pregnancy ………....1 2<br>Where to go for complication ……....1 2<br>Where to go for delivery ……………..1 2<br>How to be prepared if delivery starts 1 2<br>Delivery care ………………………....1 2<br>Newborn care ………………………...1 2<br>Feeding colostrums to the baby 1…………..2<br>Family planning ……………………....1 2<br>Rest during pregnancy……………..1 2
416.	**vki tc xHkZorh Fkh] D;k vkidks vk;ju Qksfyd xksfy;k¡@flji feyh Fkh **<br>When you were pregnant with your youngest child, were you given any iron folic tablets or syrup?	Yes ………………………………………..1<br>No ……………………………………….2<br>Don't Know ……………………………….3<br>(if 2 or 3 skip to 420)
417.	**D;k ml le; vkidks vk;ju Qksfyd xksfy;k¡ rhu eghus ;k mlls T;knk fnuksa ds fy, nh xbZ Fkh**<br>Did you receive enough iron folic tablets or syrup to last about three months or longer?	Yes ………………………………………..1<br>No ……………………………………….2<br>Don't Know ……………………………….3<br>(If 2 or 3 skip to 420)
418.	**vkius fdruk vk;ju xksyh@flji [kk;k **<br>How many of the iron folic tablets or syrup did you consume?<br>(if all skip to 423)	All ………………………………………..1<br>Most. ………………………………………..2<br>Few, ………………………………………..3<br>None ………………………………………..9
419.	Why you did not consume all tablets?<br>**vkius lkjh xksfy;ka D;ksa ugha [kkbZ **	Not necessary ……………………………..1<br>Forgot ……………………………..2<br>Stopped due to problem ……………………3<br>Other ………….. ……………………4

420.	**vki (Ukke) -------------------------------- ds le; tc xHkZorh Fkh] rks D;k vkidks vkSj vkids gksus okys cPps dks VsVul ls cpkus ds fy, ck¡g esa Vhdk (batsD'ku yxk;k x;k Fkk **<br>When you were pregnant with your youngest child, were you given an injection in the arm to prevent you and the baby from getting tetanus (use local term for tetanus)?	Yes ……………………………1<br>No ……………………………2<br>Don't Know ……………………………3<br>(if 2 or 3 skip to 501)
421.	**bl xHkkZoLFkk ds nkSjku vkidks fdruh ckj ;g Vhdk (batsD'ku) yxk;k x;k Fkk **<br>When you were pregnant with your last child, how many times did you get this injection?	Times injected ……………………………□

	Delivery	
501	**(Ukke) ------------------------------- dk tUe dgk¡ gqvk Fkk **<br>Where did you give birth to your youngest child? (For answers 21-31, skip to 505)<br>If source is hospital or clinic, write the name of the place. Probe to identify the type of place and circle the appropriate code.<br><br>**vxj mRrj 21&31 gks rks 505 ij tk,a**	Your home ……………………………11<br>Parents' home ……………………………12<br>Other home ……………………………13<br><br>**Public Medical Sector**<br>CHC/PHC ……………………………21<br>Sub-Centre ……………………………22<br>NGO/Trust Hospital/Clinic ……………………………23<br><br>**Private Medical Sector**<br>Pvt. hospital/Clinic ……………………………31<br><br>Other……………………………99<br>(specify)
502	**dkSu lk eq[; dkj.k Fkk ftldh otg ls vki izlo ds fy, LokLF; lqfo/kk esa ugha xbZ **<br>What is the main reason you did not go to a health facility for delivery?	Not necessary ……………………………01<br>Not customary ……………………………02<br>Cost too much ……………………………03<br>Too far/no transport ……………………………04<br>Poor quality service ……………………………05<br>No time to go ……………………………06<br>Family did not allow ……………………………07<br>Better care at home ……………………………08<br>Lack of knowledge ……………………………09<br>Nobody to accompany……………………………10<br>Other …………(specify) ……………………………99
503	**cPps dk tUe dgk¡ gks] bldk fu.kZ; fdlus fy;k**<br><br>Who mainly made the decision where to give birth?	Respondent ……………………………01<br>Husband ……………………………02<br>In-Laws ……………………………03<br>Parents ……………………………04<br>Sister/Sister-In-Law ……………………………05<br>Other member in husband's family ……………06<br>Other member in respondent's family……. ….07<br>Relatives ……………………………08<br>Friend/Neighbour ……………………………09<br>TBA/Field Worker/DAI ……………………………10<br>Other …………(specify) ……………………………99
504	**izlo ds le; fdlus lgk;rk dh Fkh **<br><br>Who assisted with the delivery of your youngest child?	(For answers A, B, or C skip to XXXX)<br><br>**Health Professional**<br>Doctor ……………………………A<br>ANM/Nurse/Midwife/LHV ……………………………B<br>Other Health Professional (specify) ………………C

<table>
<tr><td></td><td>Anyone else?

Probe for the type of person and record all persons who assisted.</td><td>Other Person
DAI (TBA) ………………………………D
Friend/Relative ………………………………E
Husband ………………………………F
Other (specify) ………………………………X
No one ………………………………Y</td></tr>
<tr><td>505</td><td>D;k eq[; dkj.k gS ftlds dkj.k vki fdlh LokLF; fo'ks"kK dh lgk;rk ugha yh \

What is the main reason you did not take the help of a health professional?</td><td>Not necessary ………………………………01
Not customary ………………………………02
Cost too much ………………………………03
Too far/no transport ………………………………04
Professional not available ………………………………05
No confidence in available profession ……….06
No time to get help ………………………………07
Family did not allow ………………………………08
Other …………(specify) ………………………………99</td></tr>
<tr><td>506</td><td>izlo ihM+k fdruh nsj rd pyh Fkh \
How long did the labour last?</td><td>Hours ………………………………□□</td></tr>
<tr><td>507</td><td>izlo ds le; dksbZ tfVyrk vkbZ Fkh \ D;k \
Were there any complications during labour/delivery?

List all that apply.</td><td><table><tr><td>Problem</td><td>YES</td><td>NO</td></tr><tr><td>Excessive bleeding</td><td>1</td><td>2</td></tr><tr><td>Tears/lacerations</td><td>1</td><td>2</td></tr><tr><td>Long labour</td><td>1</td><td>2</td></tr><tr><td>Delayed expulsion of placenta (more than 1/2 hr)</td><td>1</td><td>2</td></tr><tr><td>No complications</td><td>1</td><td>2</td></tr></table>(If no complications skip to 5XX)</td></tr>
<tr><td>508</td><td>Did you seek treatment for this?
D;k vkius fpfdRlk djkbZ \</td><td>Yes ………………………………1
No ………………………………2
(skip to 5XX)</td></tr>
<tr><td>509</td><td>Where did you go?

fpfdRlk ds fy, vki dgka xbZ Fkh \</td><td>Public Medical Sector
Govt. Hospital ………………………………11
CHC/PHC ………………………………12
Sub-Centre ………………………………13
NGO/Trust Hospital/Clinic………………………………14
NGO mobile clinick ………………………………15

Private Medical Sector
Pvt. hospital/Clinic/Maternity Home…………..21
Pvt. Mobile Clinic………………………………22
Pharmacy/Drugstore………………………………23
Other Private Sector Health Facility…………..24
Other …………(specify) ………………………………99</td></tr>
<tr><td>510</td><td>ml lqfo/kk esa vki dSls x;h Fkha \
How did you reach the facility?
(Record All)</td><td>On foot ………………………………A
By private vehicle ………………………………B
By government vehicle ………………………………C
Other …………(specify) ………………………………X</td></tr>
</table>

<table>
<tr><td>511</td><td></td><td>(Ukke) ------------------------------ ds tUe ds fdrus le; ckn vkius mls Lruiku djk;k \
How long after birth did you first put (NAME) to the breast?

IF LESS THAN HALF AN HOUR, CIRCLE ‘000'.
IF LESS THAN 24 HOURS, RECORD HOURS.
OTHERWISE, RECORD DAYS.</td><td>IMMEDIATELY/ WITHIN HALF AN HOUR .

HOURS . 1
DAYS . 2</td></tr>
</table>

512	D;k vkius Lru dks f'k'kq ls yxkus ds iwoZ igyk nw/k fupksM+k Fkk \ Did you discard out the milk from the breast before you first put your youngest child to the breast?	Yes-------------------1<br>No---------------------2
513	D;k tUe ds le; f'k'kq dks dksbZ LokLF; leL;k Fkh \ Was the baby suffering from any complications at birth?	Yes ……………………….........1<br>No ……………………………...... 2<br>(If no, skip to 601)
514	D;k leL;k Fkh \ Which complications?<br>vk¡[k yky<br>ihyk is'kkc@peZ<br>conjunctiva / Yellowish colouration of urine / skin<br>lkal esa fnDdr<br>Difficulty in breathing<br>cq[kkj<br>Fever<br>pwlus esa fnDdr<br>Fever + refusal to suck	Yes No<br>Conjunctiva 1 2<br>Breathing 1 2<br>Fever 1 2<br>Fever + Refusal 1 2<br>Other (specify)

	**Postpartum Care**<br>**Now I would like to ask you about the 1-month period after the delivery of your youngest child.**	
601	cPps ds tUe ds nks eghus ckn rd D;k vkids cPps ;k vkids LokLF; dh tk¡p fdlh LokLF; fo'ks"kK }kjk dh xbZ \ During that period, did a doctor or other health professional check your health or the health of your baby?	Yes ………………………………………….1<br>No ………………………………………….2<br>(skip to 606)
602	vkids lcls NksVs cPps ds tUe ds fdrus fnu ckn igyh tk¡p dh xbZ Fkh \ How soon after the birth of your youngest child did you first get a check-up?	Same day of birth………………………… ☐☐<br>At Day ……………………………………… ☐☐<br>In Week………………………………….. ☐☐
603	;s tk¡p dgk¡ dh xbZ Fkh \ Where did you get the check-up?<br><br>If source is hospital or clinic, write the name of the place. Probe to identify the type of place and circle the appropriate code.	Home Visit ……………………………………10<br><br>**Public Medical Sector**<br>Govt. Hospital ……………………….11<br>CHC/ PHC ……………………….12<br>Sub-Centre ……………………………………13<br>NGO/Trust Hospital/Clinic………………………14<br>NGO mobile clinic………………………………..15<br><br>**Private Medical Sector**<br>Pvt. hospital/Clinic/Maternity Home………….21<br>Pvt. Mobile Clinic……………………………….22<br>Pharmacy/Drugstore……………………………23<br>Other Private Sector Health Facility………….24<br>Other …………(specify)………………………….99
604	D;k buesa ls dksbZ tk¡p fd;k x;k Fkk \ Did any of the following happen when you had the check-up:<br>Was your abdomen examined?<br>Did you receive advice on family planning?	YES NO<br>Abdomen ………………….. 1 2<br>FP …………………………… 1 2<br>Breastfeeding ………………… 1 2<br>Baby care ………………….. 1 2

	Did you receive advice on breastfeeding?<br>Did you receive advice on baby care?<br>Did you receive advice on diet?	Diet……………………………1 2
605	**D;k igys ls lIrkg esa vkidk dksbZ vfrfjDr tk¡p fd;k x;k Fkk **<br>Did you receive any second check up within the first week?	Yes ....……………1<br>No ....……………2
606	**izlo ds nks ekg ds vUnj D;k vkidks buesa ls dqN leL;k gqbZ Fkh **<br>At any time during the first 6 weeks after the delivery of your youngest child, did you have any of the following:	**YES** **NO**<br>Massive vaginal bleeding? 1 2<br>Very high fever? 1 2
607	**D;k vkius fpfdRlk djkbZ Fkh **<br>Did you seek treatment?	Yes ………………………………………………1<br>No ………………………………………………2
608	**;s tk¡p dgk¡ dh xbZ Fkh **<br>Where did you get the treatment?	**Public Medical Sector**<br>Govt. Hospital ....………..11<br>CHC/ PHC ....…………....12<br>Sub-Centre ....…………13<br>NGO/Trust Hospital/Clinic..........……………14<br>NGO mobile clinic……………………………..15<br><br>**Private Medical Sector**<br>Pvt. hospital/Clinic/Maternity Home…………21<br>Pvt. Mobile Clinic.......................……………22<br>Pharmacy/Drugstore...................…………23<br>Other Private Sector Health Facility…………24<br>Other …………(specify) 99

609	**D;k vkius Lru dks f'k'kq ls yxkus ds iwoZ igyk nw/k fupksM+k Fkk **<br>Did you discard out the milk from the breast before you first put your youngest child to the breast?	Yes------------------1<br>No--------------------2
610	In the first three days after delivery, was (NAME) given anything to drink other than breast milk?	**YES** .<br>NO .
	What was (NAME) given to drink?<br><br>Anything else?<br><br>RECORD ALL	MILK (OTHER THAN BREAST MILK)<br>PLAIN WATER .<br>SUGAR OR GLUCOSE WATER<br>GRIPE WATER<br>SUGAR-SALT-WATER SOLUTION<br>FRUIT JUICE<br>INFANT FORMULA<br>TEA .<br>HONEY .<br>JANAM GHUTTI<br>OTHER<br>(SP
