# Supplementary material for: “I am not telling. The mobile is telling”: Factors influencing the outcomes of a community health worker mHealth intervention in India
Source: PLoS One. 2018 Mar 27;13(3):e0194927. doi: 10.1371/journal.pone.0194927 (PMC5870994; doi:10.1371/journal.pone.0194927)
Supplement: S3 File — (PDF) [file pone.0194927.s003.pdf]

S3 File. Qualitative Guide

Sahiyya Guide

I would like to tell you a story Sunita a Sahiyya and her experience working with a pregnant woman called Rina using a mobile phone. I will tell you part of the story, then I would like you to help me complete the story.

Sunita is 40 years old and lives in a small village in India. She is married to Ravi and they have three healthy children. She has worked as a sahiyya for over 5 years. Two years ago an NGO approached her to be part of a program where she would use a mobile phone in her work with pregnant women. Sunita agreed and joined the program. In return she received a mobile phone with questions on it. The NGO organized some training for Sunita and other ASHA's. She enjoyed the training but still had problems operating the mobile phone well. After the training is over Sunita still has questions about mobile phone but does not ask them.

Please think for Sunita, as a fellow Sahiyya.

Why do you think Sunita became a Sahiyya?

Why did she join the mobile phone program?

Thinking of Sunita as a Sahiyya from a neighboring village, what problems do you think Sunita could have with the mobile phone?

- *Why do you think she had these problems with the phone after training?*
- *What advice would you give Sunita on how to handle her problems*

Rina lives in Sunita's village; she is 25 years old and married with 2 previous children. When she becomes pregnant Sunita, goes to visit her. They exchange greetings and small talk about their families and then Sunita begins to advise Rina about her pregnancy. Sunita talks to Rina about why she should go to the Angwadi center and attend ANC clinics. She also tells Rina about other important things to do during her pregnancy like eating enough food, getting plenty of rest and not lifting heavy things. In the months following Sunita's visit, Rina tries to follow her advice and goes to two ANC visits and then stops.

How do you think Rina feels about Sunita?

- *Do you think Rina has confidence in the information Sunita shares with her*
    - *Why or why not?*
- *Do you think Sunita is comfortable discussing all aspects of sexual and reproductive health with Rina?*
    - *Why or why not ?*

Thinking about the information Sunita will provide as a trained Sahiyya do you think Rina will follow the advice?

- *Why or why not?*
- *Whether*

Rina attended two ANC visits, what do you think about that?

- *Was this enough visits?*
- *Do women in your village go a similar amount of times? Why or why not?*

Sunita had worked with Rina during her last pregnancy, but this time the session is different, Sunita brings a mobile phone. Sunita explains to Rina that she will use to the phone to record information about Rina's pregnancy. She also uses the phone as she teaches Rina. The mobile phone has pictures and words in Hindi on it, after Sunita enters Rina's information a woman's voice speaks out of the phone with pregnancy related information.

What do you think Rina felt about Sunita entering her information into the mobile phone for saving?

How do you think Rina felt about the information provided by the mobile phone?

- *What do you think Rina felt about what she was shown?*
- *How it was shown?*

This is a picture of Sunita going to see a pregnant woman before the mobile health picture

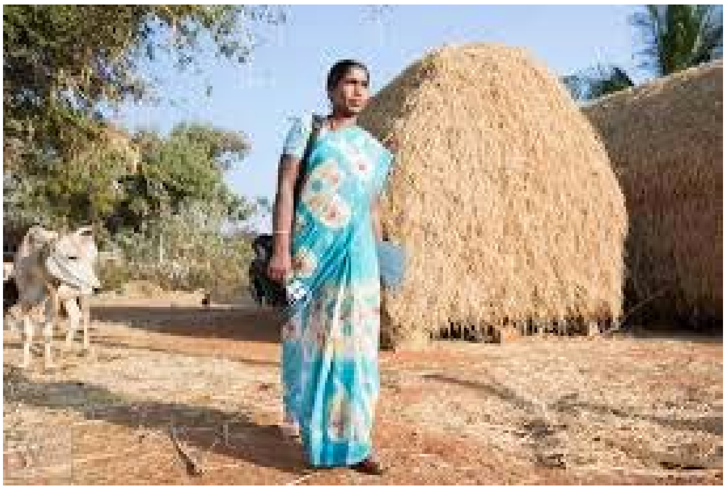

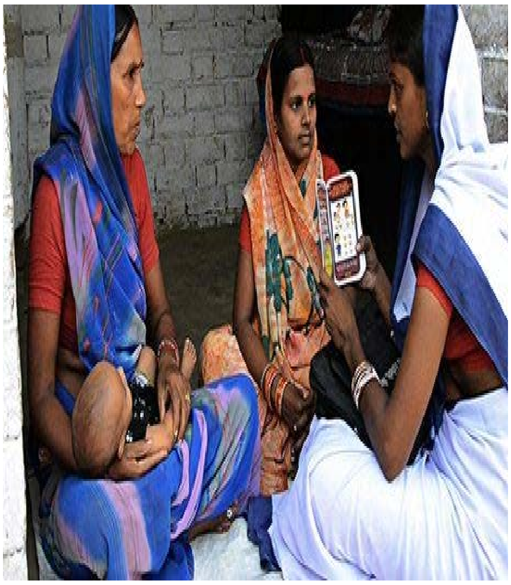

This is a picture of Sunita working with the mobile phone

How did Sunita do her work before the mobile?

How does Sunita do her work now?

- *Do you think there are any differences?*
- *Advantages, disadvantages?*
- *Any personal differences to Sunita?*
- *Any differences in the interaction between Sunita and Rina*

As the time for Rina's delivery draws closer, Sunita visits again with the mobile and emphasizes the importance of delivering the baby in the health center. She gives the woman her medications like folic acid to take during her pregnancy, also discusses options for feeding the infant, and advises Rina to breastfeed the child for 6 months without any replacement food.

Where do you think Rina will give birth to her child?

- *In your village: where do most women deliver? Why?*

I'll now tell you the next part of the story:

Although Rina wanted to deliver at the facility, she stayed at home because she could not get to the hospital.

- **Can you give reasons why Rina may not have gone to the hospital to have her baby?**

She gave birth to a healthy baby and they are doing well. Sunita visits again with the mobile phone and encourages Rina to take her child to the hospital for check-ups.

Do you think Rina take her child to the hospital?

- *Why or Why not?*
- *Why might Rina not be able to take her child to the hospital?*

Will she be able to follow the advice about breastfeeding?

- *Why/why not?*

Thinking as a Sahiyya that uses Mobile for Mothers, what do you think about Sunita and Rina's story?

- *Does it reflect what can happen in real life?*
- *Is there any information related to the events in the story you would like to share?*

Guide for Pregnant/Lactating Women and Men

Now I would like to tell you a story about a pregnant woman called Rina and her experiences being pregnant, access antenatal clinic (ANC), delivery and infant health services. I will tell you part of the story, then I would like you to help me complete the story.

Rina lives in a small village in Sarwan, she is 25 years old. She is married to Sanjay and she has 2 children. When she becomes pregnant and her village Sahiyya, Sunita, comes to visit her. Sunita comes to visit Rina with mobile phone. They exchange greetings and small talk about their families and then Sunita begins to advise Rina about her pregnancy. Sunita talks to Rina about why she should go to the Angwadi center and attend ANC clinics. She also tells Rina about other important things to do during her pregnancy like eating enough food, getting plenty of rest and not lifting heavy things. In the months following Sunita's visit, Rina tries to follow her advice and goes to two ANC visits and stops.

Please think for Rina, as a woman in your community, and imagine what she would be thinking and feeling at this time:

How do you think Rina feels about Sunita?

- *Does Rina trust Sunita?*
    - *Why or Why not*
- *How often do you think that Sunita and Rina meet?*
- *What kind of conversations do you think they have?*
- *Do you think Rina has confidence in the information Sunita shares with her*
    - *Why or why not?*

- *What types of questions or information do you think Rina is comfortable asking or sharing with Sunita?*

Do you think Rina will go to the Anagwadi center?

- *Why or Why not?*

Do you think Rina will eat the types of foods prescribed by Sunita?

- *Why or Why not?*

Rina attended two ANC visits, what do you think about that?

- *Was this enough visits?*

- *Do women in your village go a similar amount of times? Why or why not?*

Sunita had worked with Rina during her last pregnancy, but this time the session is different, Sunita brings a mobile phone. Sunita explains to Rina that she will use to the phone to record information about Rina's pregnancy. She also uses the phone as she teaches Rina. The mobile phone has pictures and words in Hindi on it, after Sunita enters Rina's information a woman's voice speaks out of the phone with pregnancy related information.

What do you think Rina felt about Sunita entering her information into the mobile phone for saving?

How do you think Rina felt about the information provided by the mobile phone?

- *What do you think Rina felt about what she was shown?*

- *How it was shown?*

- *Do you think Rina might have wanted something else on the mobile phone?*

As the time for Rina's delivery draws closer, Sunita visits again with the mobile and emphasizes the importance of delivering the baby in the health center. She gives the woman her medications like folic acid to take during her pregnancy, also discusses options for feeding the infant, and advises Rina to breastfeed the child for 6 months without any replacement food.

Where do you think Rina will give birth to her child?

- *In your village: where do most women deliver? Why?*

I'll now tell you the next part of the story:

Although Rina wanted to deliver at the facility, she stayed at home because she could not get to the hospital.

- **Can you give reasons why Rina may not have gone to the hospital to have her baby?**

She gave birth to a healthy baby and they are doing well. Sunita visits again with the mobile phone and encourages Rina to take her child to the hospital for check-ups.

Should Rina take her child to the hospital?

- *Why or Why not?*
- *Why might Rina not be able to take her child to the hospital?*

Will she be able to follow the advice about breastfeeding?

- *Why/why not?*

Does Rinas story reflect what can happen in real life?

- *Why/why not?*
